# Haemodynamic effects of plasma-expansion with hyperoncotic albumin in cirrhotic patients with renal failure: a prospective interventional study

**DOI:** 10.1186/1471-230X-8-39

**Published:** 2008-08-27

**Authors:** Andreas Umgelter, Katrin Wagner, Wolfgang Reindl, Nils Nurtsch, Wolfgang Huber, Roland M Schmid

**Affiliations:** 1 II. Medizinische Klinik, Klinikum rechts der Isar der Technischen Universität München, Ismaninger Str. 22, D-81675 München, Germany; 2 Klinik für Kardiologie, Städtisches Klinikum Bogenhausen, Englschalkinger Str. 77, D-81925 München, Germany

## Abstract

**Background:**

Patients with advanced cirrhosis of the liver typically display circulatory disturbance. Haemodynamic management may be critical for avoiding and treating functional renal failure in such patients. This study investigated the effects of plasma expansion with hyperoncotic albumin solution and the role of static haemodynamic parameters in predicting volume responsiveness in patients with advanced cirrhosis.

**Methods:**

Patients with advanced cirrhosis (Child B and C) of the liver receiving albumin substitution because of renal compromise were studied using trans-pulmonary thermodilution. Paired measurements before and after two infusions of 200 ml of 20% albumin per patient were recorded and standard haemodynamic parameters such as central venous pressure (CVP), mean arterial pressure (MAP), systemic vascular resistance index (SVRI), cardiac index (CI) and derived variables were assessed, including global end-diastolic blood volume index (GEDVI), a parameter that reflects central blood volume

**Results:**

100 measurements in 50 patients (33 m/17 w; age 56 years (± 8); Child-Pugh-score 12 (± 2), serum creatinine 256 μmol (± 150) were analyzed. Baseline values suggested decreased central blood volumes GEDVI = 675 ml/m^2 ^(± 138) despite CVP within the normal range (11 mmHg (± 5). After infusion, GEDVI, CI and CVP increased (682 ml/m^2 ^(± 128) vs. 744 ml/m^2 ^(± 171), p < 0.001; 4.3 L/min/m^2 ^(± 1.1) vs. 4.7 L/min/m^2 ^(± 1.1), p < 0.001; 12 mmHg (± 6) vs. 14 mmHg (± 6), p < 0.001 respectively) and systemic vascular resistance decreased (1760 dyn s/cm^5^/m^2 ^(± 1144) vs. 1490 dyn s/cm^5^/m^2 ^(± 837); p < 0.001). Changes in GEDVI, but not CVP, correlated with changes in CI (r^2 ^= 0.51; p < 0.001). To assess the value of static haemodynamic parameters at baseline in predicting an increase in CI of 10%, receiver-operating-characteristic curves were constructed. The areas under the curve were 0.766 (p < 0.001) for SVRI, 0.723 (p < 0.001) for CI, 0.652 (p = 0.010) for CVP and 0.616 (p = 0.050) for GEDVI.

**Conclusion:**

In a substantial proportion of patients with advanced cirrhosis, plasma expansion results in an increase in central blood volume. GEDVI but not CVP behaves as an indicator of cardiac preload, whereas high baseline SVRI is predictive of fluid responsiveness.

## Background

Patients with advanced cirrhosis of the liver characteristically suffer from circulatory disturbance [1]. Portal hypertension leads to mesenteric vasodilation. Peripheral vascular resistance is decreased and a hyperdynamic circulation ensues. Due to the pooling of blood in the splanchnic vessels, central blood volume is diminished [2] and endogenous vasopressor systems are activated in compensation [3]. These patients are vulnerable to further haemodynamic insults and, if renal auto-regulation is overwhelmed, acute kidney failure is a common complication [4]. This is termed hepatorenal syndrome (HRS), if renal failure is advanced (serum creatinine > 133 μmol/l) and if septic shock and prerenal azotaemia caused by volume losses are excluded. It negatively affects mortality [5], even if transplantation is performed [6].

The maintenance of a stable circulation is therefore important in cirrhotic patients. Plasma expansion with albumin has been found to protect against renal failure in spontaneous bacterial peritonitis [7]. It has also become a mainstay in the treatment of HRS and a prerequisite of its diagnosis, particularly when, according to a current consensus statement, "true hypovolaemia" has to be excluded by administration of a substantial amount (up to 100 g daily) of albumin over two days [8]. During acute conditions such as infection or haemorrhage, as well as for the treatment of functional renal failure, parameter guided fluid therapy may be useful for avoiding or improving relative hypovolemia, but also for avoiding potential complications of fluid overload. Data on the haemodynamic effects of an albumin infusion in cirrhotic patients, however, are scarce and it is not clear which parameters should be employed to guide fluid therapy in cirrhotic patients. Central venous pressure (CVP) has been shown to be of little value in the assessment of fluid responsiveness in critically ill patients [9-11]. In addition, elevated intra-abdominal pressure, as seen in ascitic patients, may influence CVP [12]. It therefore is of no surprise that in a recent study the traditional target values for CVP apparently failed to exclude hypovolaemia in patients with HRS[13]. Pulmonary artery catheters have traditionally been used for guiding haemodynamic interventions. Their use however remains controversial as they may be associated with complications and their predictive value in the assessment of fluid responsiveness is low [9,14-16]. Based on earlier work on indicator dilution techniques for the measurement of cardiac output (CO) and intra-thoracic blood volumes [17,18], trans-pulmonary thermodilution combined with continuous measurement of CO by pulse contour analysis has in recent years been evaluated [10,11,19]. Trans-pulmonary thermodilution has shown some promise in guiding fluid therapy by providing preload-associated parameters such as the global end-diastolic volume (GEDV). A treatment protocol targeting GEDV assessed by trans-pulmonary thermodilution resulted in reduced need for catecholamines and less time on mechanical ventilation in cardiac surgery patients [20].

It is, however, questionable if results from studies in non-cirrhotic patients are to be extrapolated to cirrhotic patients. Earlier studies found that central blood volume in cirrhotic patients cannot be significantly expanded, thus casting doubt on possible correlations between intra-thoracic blood-volumes, as a measure of preload, and CO [21,22]. To date, trans-pulmonary thermodilution has only been evaluated in ventilated cirrhotic patients during orthotopic transplantation of the liver [23].

The aim of this present study was to investigate the haemodynamic response to volume loading with hyperoncotic albumin solution, and to compare CVP and volumetric measures as markers of preload, and predictors of fluid responsiveness, in cirrhotic patients.

## Methods

The institutional ethics committee (Ethikkommission des Klinikums Rechts der Isar der Technischen Universität München) considered the protocol part of clinical routine and waived the need for informed written consent. Written consent was obtained for the publication of data.

### Patients

Patients with cirrhosis Child-Pugh-Class B or C and ascites grade II or III treated in our ICU were included. Albumin substitution was prescribed according to our internal standards because of the risk of kidney failure as defined by the RIFLE criteria (acute increase in serum creatinine of 1.5 times baseline or oliguria of < 0.5 ml/kg/h for at least 6 hours), or established renal failure along with indicators of pre-renal kidney failure (fractional excretion of sodium < 1% and no evidence of pre-existing renal impairment). To be included, patients had to already be instrumented with a thermodilution arterial line and a central venous catheter. Exclusion criteria were sepsis diagnosed by clinical criteria, and, if appropriate, chest x-rays, blood or urine cultures, and current haemorrhage. Patients receiving vasoactive or cardiotropic drugs were also excluded, as were mechanically ventilated patients.

Albumin substitution was prescribed according to our treatment standards for cirrhotic patients with pre-renal kidney failure as suggested by the recent literature [8].

### Haemodynamic Measurements

Patients were studied in a supine position, with zero pressure at the midaxillary line. CVP was recorded at end-expiration; thermodilution measurements using 15 ml of ice-cold saline were recorded in triplicate using a commercially available device (PiCCO; Pulsion Medical Systems, Munich, Germany) and their average recorded. Briefly, transpulmonary thermodilution works when a bolus of cold saline solution is injected via a central line (usually located in the superior vena cava) and detected downstream by a thermistor at the tip of the femoral arterial catheter. CO is calculated by the analysis of the thermodilution curve using the Stewart-Hamilton algorithm. Mean transit time and exponential downslope time of the thermodilution curve are also analysed. The product of CO and mean transit time is the volume of distribution of the thermal indicator, or "intra-thoracic thermal volume" comprising of the intra-thoracic blood volume and the extravascular lung water. The product of CO and exponential downslope time is the "pulmonary thermal volume" composed of the pulmonary blood volume and the extravascular lung water. The GEDV is obtained as the difference between intra-thoracic thermal volume and pulmonary thermal volume [24].

Values for mean arterial pressure (MAP), CVP, CO, Systemic Vascular Resistance (SVR), GEDV, Stroke Volume (SV) and Heart Rate (HR) were obtained, and, when appropriate, indexed to an estimate of body surface area (BSA), according to the formula of duBois, to calculate Cardiac Index (CI), Global End-diastolic Volume Index (GEDVI), Systemic Vascular Resistance Index (SVRI) and Stroke Volume Index (SVI). Stroke volume divided by pulse pressure was used as a marker for arterial compliance and calculated as follows: compA = SV/(RRsys – RRdia) [25]. Cardiac power index was calculated as CPI = CI × MAP/451 [26].

### Study Protocol

Haemodynamic measurements were performed immediately before infusion of a bolus of 200 ml 20% albumin solution over a short time period (< 30 min). Measurement of haemodynamic variables were repeated 1 hour after the start of infusion, to allow for maximal plasma expansion. Albumin infusions were prescribed by the treating physician and were administered as aliquots of 200 ml 20% solution (40 g of albumin). For statistical reasons, only the first two measurements after inclusion for each patient were analysed.

### Statistical analysis

Data were assessed for normal distribution using the Kolmogorov-Smirnov test. Normal distribution was found for all haemodynamic parameters. Accordingly, data are presented as mean (± SD). Haemodynamic parameters before and after albumin infusion were compared using Students T-test for paired samples.

Parameters displaying significant changes in univariate analysis were evaluated for correlations with Pearson's test.

A positive response to volume loading was defined as an increase in CI of > 10%. This value was chosen for the following reasons: Previous studies investigating fluid responsiveness have used cut-off values for CI of 10–20% [16]. Reproducibility of measurements of CO and GEDV have been found to be 4 ± 2% and 5 ± 2%, respectively, and increases after plasma expansion of an amount similar to our study previously resulted in mean increases in cardiac output of 7 – 15% [22].

Responders and non-responders were compared using the Student T test for unpaired samples. For baseline haemodynamic parameters that were significantly different between responders and non-responders, ROC curves were constructed to analyse their respective value to predict an increase of CI by >10% after volume loading.

## Results

### Baseline parameters

50 consecutive patients were included between August 2005 and January 2007. Baseline characteristics are presented in Table 1. Significant correlations were found between baseline values of GEDVI and CI (r^2 ^= 0.20; p = 0.001) and between baseline values of GEDVI and SVI (r^2 ^= 0.28; p < 0.001).

**Table 1:** Baseline characteristics of all patients

Age (years)	56 (± 8)
Gender (m/f)	33/17
MELD-score	28 (± 9)
Child-Pugh-score	12 (± 2)
Child-Pugh class (B/C)	10/40
Serum creatinine (μmol/l)	256 (± 150)
Fractional excretion of Sodium (%)	0.040 (± 0.026)
Creatinine clearance (ml/min)	22 (± 16)
CVP (mmHg)	11 (± 5)
GEDVI (ml/m^2^); (n: 680–800)	675 (± 138)
CI (L/min/m^2^); (n: 3–5)	4.1 (± 1.2)
SVI (ml/m^2^); (n: 40–60)	48 (± 13)
HR (bpm)	88 (± 20)
SVRI (dyn s/cm^5^/m^2^); (n:1700–2400)	1898 (± 1015)
comp_a _(ml/mmHg)	1.58 (± 0.57)
MAP (mmHg)	79 (± 14)
CPI (mmHg L/min/m^2^)	0.71 (± 0.27)

MELD: Model of End-Stage Liver Disease; CVP: Central Venous Pressure; GEDVI: Global End-Diastolic Volume Index; CI: Cardiac Index; SVI: Stroke Volume Index; HR: Heart Rate; SVRI: Systemic Vascular Resistance Index; comp_a_: Arterial Compliance; MAP: Mean Arterial Pressure; CPI: Cardiac Power Index

### Haemodynamic effects of fluid loading

Haemodynamic parameters obtained before and after 100 albumin infusions were analysed and are presented in Table 2. In 43 cases there was an increase in CI of > 10% after albumin infusion.

**Table 2:** Haemodynamic parameters before and after infusion of 200 ml of 20% albumin solution.

	Before	after	
CVP (mmHg)	12 (± 6)	14 (± 6)	p < 0.001 95% CI 1 – 3
GEDVI (ml/m^2^); (n: 680–800)	682 (± 128)	744 (± 171)	p < 0.001 95% CI 38 – 87
CI (L/min/m^2^); (n: 3–5)	4.3 (± 1.1)	4.7 (± 1.1)	p < 0.001 95% CI 0.3 – 0.5
SVI (ml/m^2^); (n: 40–60)	49 (± 12)	54 (± 13)	p < 0.001 95% CI 2 – 6
HR (bpm)	89 (± 18)	90 (± 16)	p = 0.816 95% CI -3 – 1
SVRI (dyn s/cm^5^/m^2^); (n: 1700–2400)	1760 (± 1144)	1490 (± 837)	p < 0.001 95% CI -370 – -170
compA (ml/mmHg)	1.59 (± 0.52)	1.70 (± 0.62)	p = 0.040 95% CI -0.21 – 0.02
MAP (mmHg)	78 (± 12)	80 (± 13)	p = 0.310 95% CI -1 – 4
CPI (mmHg L/min/m^2^)	0.72 (± 0.27)	0.81 (± 0.31)	p < 0.001 95% CI 0.06 – 0.13

CVP: Central Venous Pressure; GEDVI: Global End-Diastolic Volume Index; CI: Cardiac Index; SVI: Stroke Volume Index; HR: Heart Rate; SVRI: Systemic Vascular Resistance Index; comp_a_: Arterial Compliance; MAP: Mean Arterial Pressure; CPI: Cardiac Power Index

### Correlations between changes in haemodynamic parameters

Changes in CI following volume challenges showed significant correlations with changes in GEDVI (r^2 ^= 0.51; p < 0.001), but not with changes in CVP (r^2 ^0.01, p = 0.45) or MAP (r^2 ^= 0.01; p = 0.26). Likewise, changes in SVI were correlated with changes in GEDVI (r^2 ^= 0.27; p < 0.001) but not with changes in CVP or MAP. As expected, there was an inverse correlation between CI and SVRI (r^2 ^= 0.21, p < 0.001), but no correlation between CI or GEDVI and MAP.

### Predictors of fluid responsiveness

In responders CVP, GEDVI, SVI and CI were significantly lower than in non-responders, whereas SVRI was significantly higher (Table 3).

**Table 3:** Differences in pre-infusion haemodynamic parameters between responders and non-responders

	responders	non-responders	
CVP (mmHg)	10 (± 4)	13 (± 6)	p < 0.001 95% CI 1 – 3
GEDVI (ml/m^2^); (n: 680–800)	638 (± 135)	714 (± 117)	p < 0.001 95% CI 38 – 87
CI (L/min/m^2^); (n: 3–5)	3.7 (± 1.0)	4.7 (± 1.0)	p < 0.001 95% CI 0.3 – 0.5
SVI (ml/m^2^); (n: 40–60)	45 (± 14)	54 (± 9)	p < 0.001 95% CI 2 – 6
HR (bpm)	90 (± 15)	86 (± 19)	p = 0.816 95% CI -3 – 1
SVRI (dyn s/cm^5^/m^2^); (n:1700–2400)	2262 (± 1323)	1390 (± 648)	p < 0.001 95% CI -370 – -170
compA (ml/mmHg)	1.49 (±	1.68 (± 0.56)	p = 0.040 95% CI -0.20 – 0.02
MAP (mmHg)	78 (± 13)	80 (± 13)	p = 0.310 95% CI -1 – 4
CPI (mmHg L/min/m^2^)	0.71 (± 0.27)	0.81 (± 0.32)	p < 0.001 95% CI 0.06 – 0.12

CVP: Central Venous Pressure; GEDVI: Global End-Diastolic Volume Index; CI: Cardiac Index; SVI: Stroke Volume Index; HR: Heart Rate; SVRI: Systemic Vascular Resistance Index; comp_a_: Arterial Compliance; MAP: Mean Arterial Pressure; CPI: Cardiac Power Index

**Figure 1:**
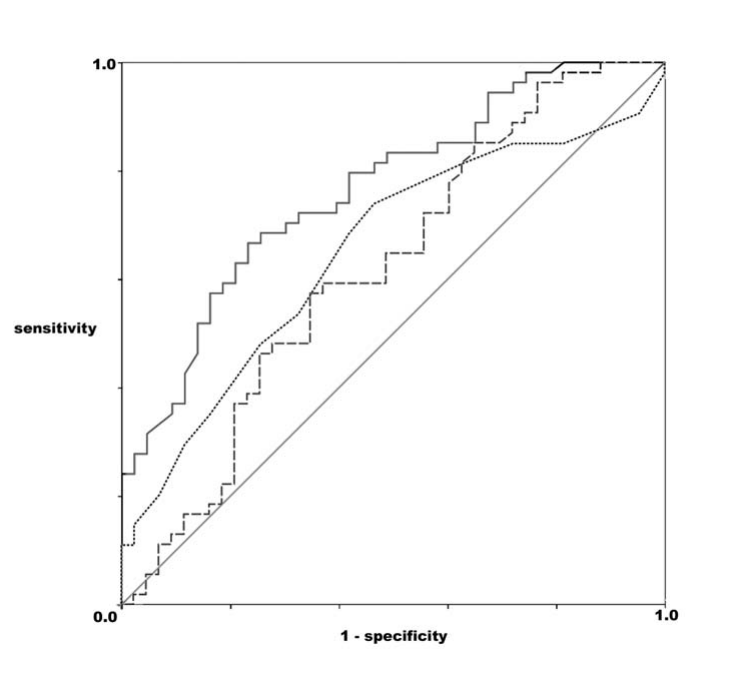
**Receiver operating characteristic curves for systemic vascular resistance index (continuous line), central venous pressure (dotted line) and global and-diastolic volume index (broken line).** The diagonal line is the line of no-discrimination.

ROC-Curves for GEDVI, CVP and SVRI are displayed in Figure 1. Area under the curve was greatest for SVRI (area 0.766; p < 0.001, 95%CI 0.674 – 0.859) and CI (area 0.723; p < 0.001, 95%CI 0.629 – 0.816), still significantly better than chance (area = 0.5) for CVP (area 0.652; p = 0.010, 95%CI 0.542 – 0.761) and bordering significance for GEDVI (area 0.616; p = 0.050), 95%CI 0.501 – 0.731). For SVRI a cut-off value of 1270 dyne·s/cm^5^/m^2 ^discriminated between responders and non-responders (sensitivity 0.67, specificity 0.77). For CVP the best combined sensitivity and specificity was found for a threshold value of 10 mmHg with 0.74 and 0.54, respectively. For GEDVI a threshold value of 680 ml/m^2 ^had a sensitivity of 0.59 and a specificity of 0.65 for predicting a positive response to albumin infusion.

## Discussion

At baseline we found a GEDVI in the lower range of normal despite a relatively high CVP. CI was in the normal range and SVRI at a low normal value. These findings are in accordance with the concept suggested by the peripheral arterial vasodilation hypothesis on cirrhotic circulatory dysfunction [1]. Infusion of albumin solution resulted in an increase in GEDVI, which correlated to an increase in cardiac index that was larger than 10% in almost half the cases. This is similar to what has previously been reported in fluid resuscitation of septic patients [11]. Responders to volume loading displayed a baseline haemodynamic pattern suggestive of lower cardiac preload with less hyperdynamic circulation and higher peripheral resistance.

The improvement in CI after volume therapy supports the notion that relative central hypovolaemia contributes to circulatory dysfunction in cirrhotic patients. After plasma expansion with albumin we found an increase in central blood volume. This is in contrast to the results of other studies who failed to detect relevant changes in central blood volume after fluid loading in patients with advanced cirrhosis [22,27]. We believe that in these studies the possible effects were missed due to the small number of patients included. Indeed, both studies showed increases in central blood volume after volume loading, however, this failed to reach statistical significance.

In our study, baseline GEDVI correlated with baseline CI and SVI, and GEDVI was lower in patients with a positive response to volume loading than in those with a negative response. Furthermore, increases in GEDVI correlated to increases in CI and SVI, highlighting that GEDVI, evaluated by trans-pulmonary thermodilution, behaves as an indicator of preload in patients with cirrhosis. CVP was lower in patients who responded to volume loading and increased significantly after infusion. The volume loading-induced changes, however, were not proportional to the changes in CI and SVI, and baseline CVP did not correlate with CI or SVI. This confirms previous reports and underlines the limited value of CVP as a marker of cardiac preload. Both parameters performed poorly as predictors of volume responsiveness as has been previously documented in various clinical settings [16,28].

Cardiac preload is defined as myocardial wall tension at end diastole, and, according to Laplace's law, is determined by ventricular geometry and intra-ventricular pressures. Myocardial contractility depends on end-diastolic tension of the myocardial sarcomers and the connection between increasing preload and contractility is given in the sigmoidal Frank-Starling curve. Without knowing the individual myocardial properties at the moment of interest, we cannot determine the position on the Frank-Starling curve of any given preload condition. This explains why good intra-individual correlations between preload markers and CI in paired measurements may be accompanied by a low predictive value of single measurements of preload associated markers for fluid responsiveness.

Following volume challenges we observed substantial decreases in SVRI in our patients. SVRI is dependant on (MAP-CVP) and CI by a linear relationship. Therefore, with constant CVP, any changes in CI must be accompanied by proportional changes in SVRI, MAP, or both. In patients with septic shock, opposite changes of a similar relative size of both MAP and SVRI have been observed after volume loading [11]. In contrast, we found large decreases in SVRI with only minuscule increases in MAP. This contrasts to a previous study on plasma expansion in patients with spontaneous bacterial peritonitis (SBP) [29]. Here the authors described an increase in peripheral vascular resistance after treatment of SBP with antibiotics and albumin. They hypothesized that this may be due to the pharmacological action of albumin as a scavenger of nitric oxide, thus reducing the vasodilatory properties of plasma. However, haemodynamic measurements in this study were days apart and the increased vasotonus, may have been due to reduced septic vasodilation. In our study cohort, care was taken to select patients without infection or haemorrhage, so that any related confounding factors were avoided.

Whereas MAP was not different between the patients who responded to volume loading and those who did not, SVRI was significantly and by a large proportion higher in responders when compared to non-responders. However, CI was significantly lower in responders than in non-responders. Pre-infusion values of SVRI (and CI) were predictive of volume responsiveness in our patients. As suggested previously [30], this may indicate that in a proportion of patients with cirrhotic circulatory dysfunction, relative central hypovolaemia, resulting in further activation of endogenous vasopressor systems to maintain MAP at the cost of high peripheral resistance, may contribute to impaired cardiac output, despite what is essentially a hyperdynamic circulation. Volume therapy may thus decrease vasopressor activation, and may lead to decreased levels of endogenous vasopressors such as norepinephrine, renin and angiotensin, as has been described previously [22]. Renal dysfunction in cirrhosis deteriorates along a continuum starting with an impaired capacity to excrete sodium and free water leading to an oedematous state with increased plasma volume and ascites, to pre-renal failure and, finally, irreversible tubular damage. According to current understanding, an important etiologic factor is elevated levels of vasoconstrictors affecting the renal microcirculation, narrowing the kidneys' capacity to cope with additional haemodynamic insults. Volume management may be relevant to the prevention and treatment of functional renal failure in cirrhosis. Recent studies on vasopressor therapy in HRS highlight the importance of adequate volume status. Whereas it had previously been shown that albumin was necessary for the beneficial effect of terlipressin [31], a recent study by Alessandria et al. showed that a substantial number of patients included in a study on treatment of HRS responded to plasma expansion alone when it was tailored according to CVP instead of using the usual fixed-dose regimen [13]. In this study the aim was a CVP of 10 – 15 mmHg. In our study, 33% of patients with a CVP greater than 10 mmHg and 24% of patients a CVP of over > 15 mmHg still responded to albumin infusion with a further increase in CI. Consequently, neither CVP nor GEDVI should be recommended as parameters to direct fluid resuscitation in cirrhotic patients with pre-renal kidney failure.

In ventilated patients, dynamic parameters such as pulse pressure variation or stroke volume variation have shown much better predictive power for assessing fluid responsiveness [28]. However, the majority of cirrhotic patients at risk of renal failure are breathing spontaneously and these circumstances, dynamic parameters are not applicable. A time honoured method for the assessment of fluid responsiveness, "passive leg raising" (PLR) [32], has recently gained renewed interest in the intensive care setting. PLR generates a transient increase in venous return. The immediate haemodynamic response of mean blood flow to this manoeuvre, assessed by methods such as oesophageal Doppler [33] or trans-thoracic echocardiography [34], has been used to estimate fluid responsiveness. The recently published method of PLR is difficult to apply in the intensive care setting, because a fixed angle of the hips is required throughout the procedure and the whole bed must be tilted instantaneously by 45°. Cirrhotic patients may react differently to tilting than other patients or normal controls [35], and the elevated intra-abdominal pressure of ascitic patients may also affect PLR-induced blood transfer [36]. Therefore, PLR may give different results in cirrhotic patients. This has not to our knowledge been evaluated.

Without static parameters predictive of fluid responsiveness, but a variety of monitoring tools capable of providing data on CI and MAP, iterative protocols of fluid challenges may offer the possibility of increasing cardiac output in patients with reduced effective intravascular volume [14]. Whether this translates to improved kidney function in cirrhotic patients with renal failure should be evaluated in future studies.

The obvious limitations of our study are the uncontrolled design and the use of albumin solution instead of crystalloid solutions for the volume challenge. Hyperoncotic albumin solution acts as a plasma expander and, in addition, has distinct pharmacological properties.

## Conclusion

In contrast to earlier studies we have observed a significant increase in central blood volume and CI after albumin infusion in a substantial proportion of patients with advanced cirrhosis. In contrast to CVP, GEDVI behaved as a preload indicator, but neither parameter was able to predict fluid responsiveness with acceptable accuracy. After albumin infusion there were no relevant changes in MAP, but large decreases in SVRI. Pre-infusion SVRI and CI discriminated between patients with and without a positive response in CI to volume loading. Circulatory dysfunction in cirrhotic patients with pre-renal kidney failure may be amenable to plasma expansion and future trials to evaluate fluid resuscitation strategies in these patients are warranted.

## Competing interests

WH and AU have been invited speakers for Pulsion Medical Systems, Munich; the other authors declare no competing interests. No grants or external funding were received for this study.

## Authors' contributions

AU, KW and WR: Idea and design of the study. AU, KW, NN, WR: Collection and analysis of data. AU wrote the manuscript. WH and RMS revised and co-wrote the manuscript.

## Pre-publication history

The pre-publication history for this paper can be accessed here:


